# Gene‐Guided Therapy in Type 2 Diabetes Mellitus: An Integrative Review of the Pharmacogenetics of Metformin and Sodium‐Glucose Cotransporter‐2 Inhibitors

**DOI:** 10.1002/edm2.70343

**Published:** 2026-10-12

**Authors:** Imran Hossain, Mahnoor Haidar, Mahfuj Ahmed Ratul, Jannatul Ferdous Sumona, Sraboni Shabnaz, Abul Fojal Nokib, Fatema‐Tuz‐Zohora

**Affiliations:** ^1^ Department of Pharmacy University of Asia Pacific Dhaka Bangladesh; ^2^ Department of Clinical Pharmacy and Pharmacology, Faculty of Pharmacy University of Dhaka Dhaka Bangladesh

**Keywords:** genetic polymorphism, metformin, pharmacogenomics, precision medicine, sodium‐glucose cotransporter‐2 inhibitors, type 2 diabetes mellitus

## Abstract

**Background:**

Inter‐individual variability in the therapeutic response to antidiabetic drugs is still a big problem in the management of type 2 diabetes mellitus (T2DM). Genetic polymorphisms of drug transporters, metabolizing enzymes and molecular targets have become significant factors in drug efficacy, pharmacokinetics and adverse effects. This review aims to provide a summary of the current evidence on the pharmacogenomics of metformin and sodium‐glucose cotransporter‐2 (SGLT2) inhibitors and their implications for precision medicine in T2DM.

**Methods:**

Published human studies examining genetic polymorphisms related to the various effects of metformin and SGLT2 inhibitors in patients with T2DM were systematically searched in PubMed, Scopus and EBSCO and analysed using an integrative review. Qualitative synthesis was used to compare the pharmacogenetic profiles of the two drug classes and to look for potential biomarkers for precision therapy.

**Results:**

Metformin has the most comprehensive pharmacogenetic evidence, including variants in SLC22A1 (OCT1), SLC22A2 (OCT2), SLC22A3 (OCT3), SLC47A1 (MATE1), SLC47A2 (MATE2), ATM and other genes that affect drug disposition, glycemic response and adverse effects. However, pharmacogenetic research on SGLT2 inhibitors is still scarce. Modest variations in drug exposure, glucose homeostasis and cardiorenal or hepatic outcomes have been reported for variants of UGT1A9, UGT2B4, SLC5A2, PNPLA3, and some glucose transporters, but most of these effects have not been strong enough to recommend routine genotype‐based therapy. Metformin has more robust and consistent pharmacogenetic associations overall, while SGLT2 inhibitors have emerging but less definitive associations.

**Conclusion:**

Pharmacogenomics has great promise to enhance the selection of antidiabetic drugs, maximize therapeutic effect and minimize side effects. There is current evidence for the clinical relevance of several pharmacogenetic markers for metformin, but evidence for SGLT2 inhibitors is preliminary. Further, large, prospective, multiethnic studies and genotype‐based clinical trials are needed to confirm candidate biomarkers and to help incorporate pharmacogenomics into precision medicine for T2DM.

## Graphical abstract

Genetic variation influences the pharmacological response to metformin and SGLT2 inhibitors in patients with type 2 diabetes mellitus. Pharmacogenetic biomarkers affecting drug transport, metabolism and target activity contribute to inter‐individual differences in treatment efficacy and adverse effects, supporting the implementation of personalized therapy to optimize clinical outcomes.

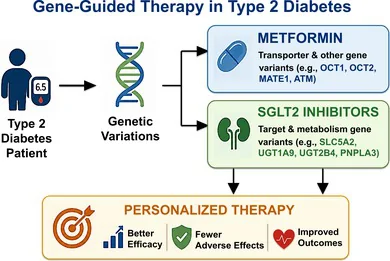

## 1 Introduction

Type 2 diabetes mellitus (T2DM) is a chronic, progressive metabolic disorder associated with chronic hyperglycemia due to insulin resistance and relative insulin deficiency, and is a significant burden of disease in the world, causing a high burden of morbidity and mortality [1]. The latest International Diabetes Federation (IDF) Diabetes Atlas indicates that over 90% of all adults (20–79 years) with diabetes have T2DM, and that there are currently an estimated 589 million people with T2DM globally, or about 1 in 9 adults, with projections of 853 million by 2050. There is a high proportion of people who are undiagnosed, with approximately 252 million adults (43% of the total diabetic population) not knowing they have diabetes, and approximately 635 million people with impaired glucose tolerance who are at higher risk of developing T2DM [2].

Although lifestyle modification is the mainstay of T2DM management, most people with T2DM need pharmacotherapy to achieve and maintain good glycemic control. Metformin is the most commonly used first‐line drug and is considered to be very effective at lowering blood glucose, has a good safety profile, and is well tolerated [3]. Another therapeutic class that has proven to be effective in lowering blood glucose levels is sodium‐glucose co‐transporter 2 (SGLT2) inhibitors, which also offer other benefits, including weight loss and cardiovascular risk reduction and mortality reduction in certain patient populations, without significantly increasing the risk of hypoglycemia [4].

Although these therapies are effective overall, there is significant inter‐individual variability in therapeutic response and tolerability, which cannot be fully explained by clinical or environmental factors [5]. This has led to an increased interest in the genetic mechanisms underlying drug response and development of polygenic tools such as polygenic risk scores (PRS) that reflect the combined effect of multiple genetic variants linked to glycemic traits, insulin resistance, β‐cell function and drug mechanisms of action. The idea of gene‐guided glycemic therapy, which is based on personalized medicine and individual genetic architecture, is gaining traction as a promising paradigm for the treatment of T2DM [6].

This review provides a critical synthesis of current knowledge on the pharmacogenomics (PGx) of metformin and SGLT2 inhibitors, explores the potential of polygenic models to predict glycemic response, and examines the ongoing shift from conventional, ‘one‐size‐fits‐all’ management toward precision medicine in T2DM. Ultimately, its aim is to help bridge the gap between genomic discovery and clinical application, facilitating more tailored and effective glycemic therapies for individuals living with T2DM.

## 2 Materials and Methods

### 2.1 Study Design

The study was designed as an integrative review to comprehensively synthesize the current evidence on the pharmacogenetics of metformin and SGLT2 inhibitors in patients with T2DM. An integrative review approach was chosen to allow for the inclusion of a variety of study designs such as pharmacogenetic association studies, pharmacokinetic studies and clinical response studies, to gain a comprehensive understanding of genetic determinants of antidiabetic drug response. The review methodology was based on the integrative review framework of Whittemore and Knafl [7] that allows for the systematic integration of evidence from a variety of methodological approaches while maintaining methodological rigour. The review was conducted in a transparent manner, and the process of selecting studies is displayed in a PRISMA 2020 flow diagram.

### 2.2 Literature Search Strategy

A literature search was conducted in PubMed, Scopus and EBSCO from database inception to July 2026 to identify relevant studies. The search strategy combined Medical Subject Headings (MeSH; where applicable), database‐specific indexing terms and free‐text keywords, including *metformin*, *SGLT2 inhibitors*, *pharmacogenetics*, *pharmacogenomics*, *genetic polymorphism*, *single nucleotide polymorphism (SNP)*, *OCT1*, *OCT2*, *OCT3*, *MATE1*, *MATE2*, *UGT1A9*, *UGT2B4*, *SLC5A2* and *type 2 diabetes mellitus*. Boolean operators (AND and OR) were used to combine and refine the search terms according to the syntax of each database. Reference lists of all eligible articles were also manually screened to identify additional relevant studies that may not have been captured through the electronic database search. The search strategy was designed to be as sensitive as possible to maximize the retrieval of potentially relevant studies while maintaining specificity. The same search concepts were applied across all databases using their respective indexing systems and search syntax.

### 2.3 Eligibility Criteria and Study Selection

Eligible articles were those published in English that examined the relationship between genetic polymorphisms and the pharmacokinetics, pharmacodynamics, therapeutic efficacy, or adverse effects of metformin or SGLT2 inhibitors in human subjects with T2DM. Reviews, meta‐analyses, editorials, conference abstracts, case reports, animal studies, in vitro studies and articles without pharmacogenetic outcomes were not included. Titles and abstracts were screened for relevance after duplicate removal and then full‐text articles were assessed based on the predetermined eligibility criteria. The final qualitative synthesis included a total of 31 original studies (18 metformin and 13 SGLT2 inhibitors; Figure 1). Due to the diversity of the studies included in the review, methodological quality was taken into account when interpreting the evidence, including study design, sample size, population characteristics and consistency of results across studies and reproducibility of results. Studies with larger sample sizes, well‐defined study designs, and findings replicated in independent populations were considered to provide more robust evidence during the narrative synthesis. Because of the heterogeneity in study designs, genetic variants, study populations, and outcome measures among the included studies, a formal risk‐of‐bias assessment was not performed, as no single validated appraisal tool was considered appropriate for all included study designs.

**FIGURE 1:**
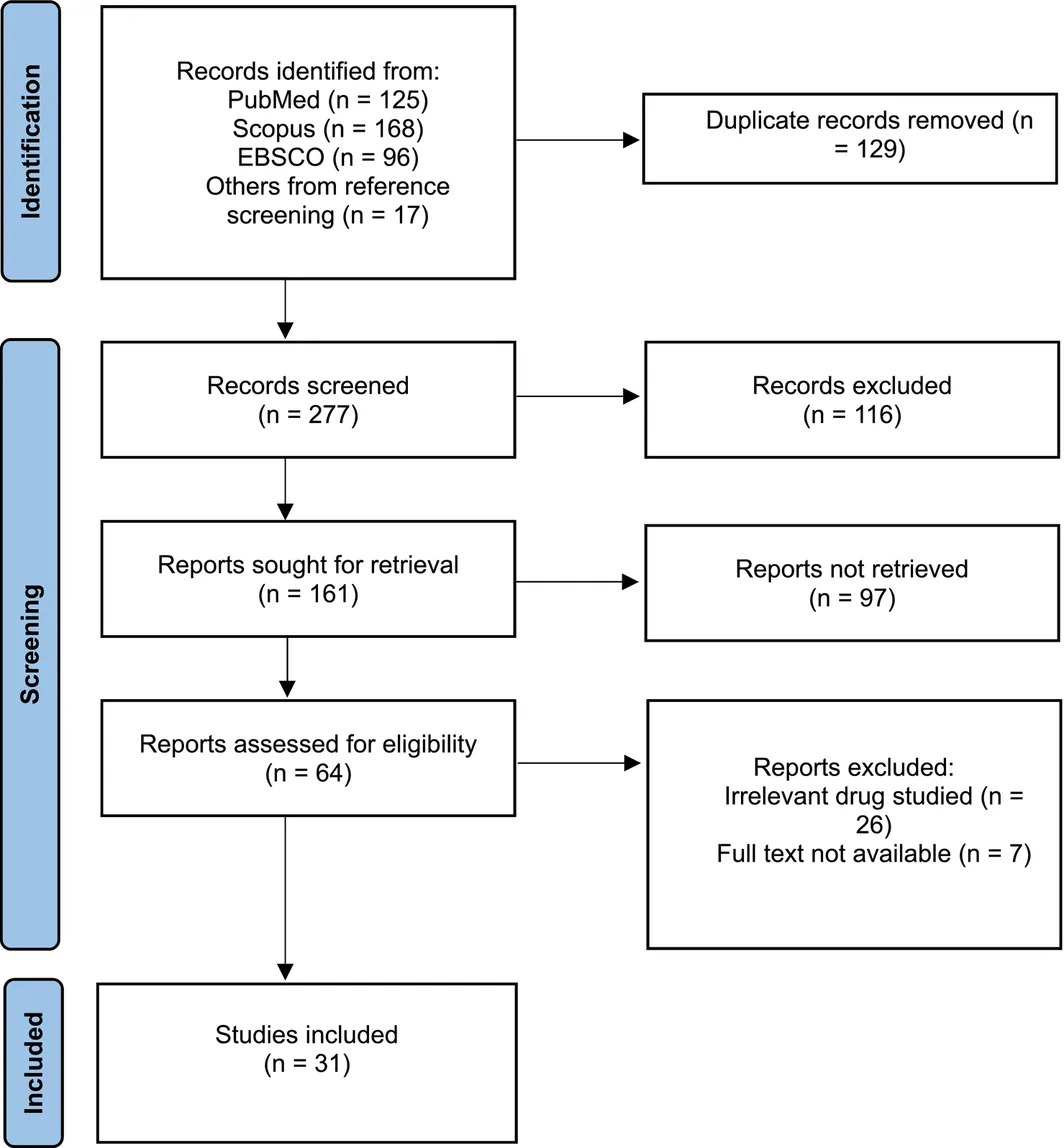
PRISMA 2020 flow diagram illustrating the study selection process for the integrative review.

### 2.4 Data Extraction and Evidence Synthesis

The characteristics of the studies, the population studied, the genes and polymorphisms investigated, the antidiabetic agents used, and the main pharmacogenetic results were retrieved from each eligible study. If available, further details on study design, sample size, ethnicity, clinical outcomes and study limitations were also collected to aid in the critical comparison of available evidence. Due to the diversity of study designs, patient populations, genetic variants studied and reported outcomes, quantitative meta‐analysis was not conducted. Instead, the results were synthesized in a narrative way and grouped by drug class, gene function, and their reported effect on pharmacokinetics, pharmacodynamics, therapeutic response and adverse effects. The strength of evidence for each pharmacogenetic association was assessed based on the consistency of the evidence from independent studies, biological plausibility and clinical relevance for precision medicine in T2DM. Associations supported by consistent findings from multiple independent studies with clear biological plausibility and potential clinical relevance were considered to represent stronger evidence, whereas associations supported by limited, conflicting, or non‐replicated findings were interpreted more cautiously.

## 3 Inter‐Individual Variability in Drug Response

One of the biggest challenges in the management of T2DM is inter‐individual variability in drug response. Patients can have significant differences in therapeutic response and side effects to the same drug and dose. This variability is due to a combination of genetic, environmental and clinical factors such as diet, lifestyle, comorbidities and concomitant medications [8]. Of these, genetic polymorphisms have become important factors in drug disposition and response, affecting the transport, metabolism and interaction of drugs with their targets. PGx aims to identify these genetic variations to enable personalized treatment and enhance treatment outcomes while reducing side effects [9].

### 3.1 Genetic Variability in Drug Metabolism

Systemic drug exposure can be affected by genetic variation in drug metabolizing enzymes, which affects metabolic capacity. People with functional variants can have higher or lower drug clearance, which can lead to differences in the effectiveness and side effects of the drug. Metformin is metabolized to a lesser extent, but glucuronidation enzymes, such as UGT1A9, are involved in the pharmacokinetics of some SGLT2 inhibitors and may impact treatment response [10].

### 3.2 Genetic Polymorphism in Drug Transporters

Drug transporters control drug absorption, tissue distribution and renal elimination. Polymorphisms in transporter genes may affect the intracellular and plasma levels of drugs and hence their therapeutic response. Polymorphisms in SLC22A1 (OCT1), SLC22A2 (OCT2) and SLC47A1/2 (MATE1/2) have been linked to variations in drug exposure, glycemic response and adverse effects for metformin [11].

### 3.3 Genetic Variability in Drug Targets

Pharmacodynamic variability is due to genetic variations in the drug target or in the downstream signalling pathways. Differences in the effects of a drug on glucose homeostasis or insulin signalling proteins can alter drug efficacy even when systemic exposure to the drug is the same. For instance, polymorphisms of IRS1 and IRS2 can affect insulin signalling and lead to decreased glycemic response to insulin‐sensitizing drugs like metformin [12, 13].

### 3.4 Polygenic Nature of Drug Response

Drug response is generally polygenic, meaning that it is the result of several genetic variants that each have a small effect. Polymorphisms in transporter genes (such as OCT1, OCT2 and MATE1/2) and metabolic enzymes and drug targets all contribute to drug exposure and therapeutic efficacy. This polygenic architecture may account for the varying effects of metformin or SGLT2 inhibitors on patients with similar clinical characteristics [14, 15].

## 4 Metformin

Metformin is a drug from the biguanide class that has been the main first‐line treatment for T2DM for more than 60 years. It remains in use today in medicine due to its effectiveness in reducing blood sugar levels and its lack of numerous side effects and its low cost for most patients [16].

Apart from glucose lowering, metformin also has other beneficial effects including mild weight loss, favourable effects on lipid profiles and possibly cardiovascular protection [17]. Its effects on cellular pathways such as AMPK and mTOR have also sparked interest in its potential applications in various health areas including cancer, polycystic ovary syndrome (PCOS), non‐alcoholic fatty liver disease (NAFLD) and aging [18]. Recent research further indicates that metformin has an effect on the gut microbiome and that it may also stimulate the production of the metabolite N‐lactoyl‐phenylalanine (Lac‐Phe), which could play a role in regulating appetite and body weight [19]. It is generally well tolerated, but common side effects include gastrointestinal symptoms like nausea and diarrhoea, and a rare risk of lactic acidosis is associated with it, which should be taken into account in patients with severe kidney impairment [20].

### 4.1 Mechanism of Action

Metformin acts on a number of complementary pathways, the primary one being the suppression of hepatic glucose production. It helps to dampen the overproduction of glucose by the liver, thereby inhibiting the action of important enzymes like phosphoenolpyruvate carboxy‐kinase (PEPCK) and glucose‐6‐phosphatase, especially in the pyruvate carboxylase‐PEPCK pathway, which slows down the process of gluconeogenesis. It also decreases the activity of cAMP and protein kinase A (PKA), which normally increase blood sugar, further reducing glucose output [21]. In addition, metformin shifts the cell's redox balance by blocking mitochondrial glycerophosphate dehydrogenase, making it harder for lactate and glycerol to be converted into glucose [16].

In addition to this, metformin stimulates AMPK, the energy sensor of the cells, by inhibiting mitochondrial complex I, thereby reducing ATP and increasing AMP. This activation lowers the production of glucose by the liver, lowers fat production, increases insulin sensitivity, and regulates genes associated with glucose metabolism (Figure 2). It also improves insulin sensitivity by increasing the uptake of glucose in muscle and fat cells, improving insulin signalling, and increasing the activity of GLUT 4, which facilitates the entry of glucose into cells more effectively. These effects are further strengthened by fat breakdown and fat reduction, which help to improve insulin sensitivity [18, 21].

**FIGURE 2:**
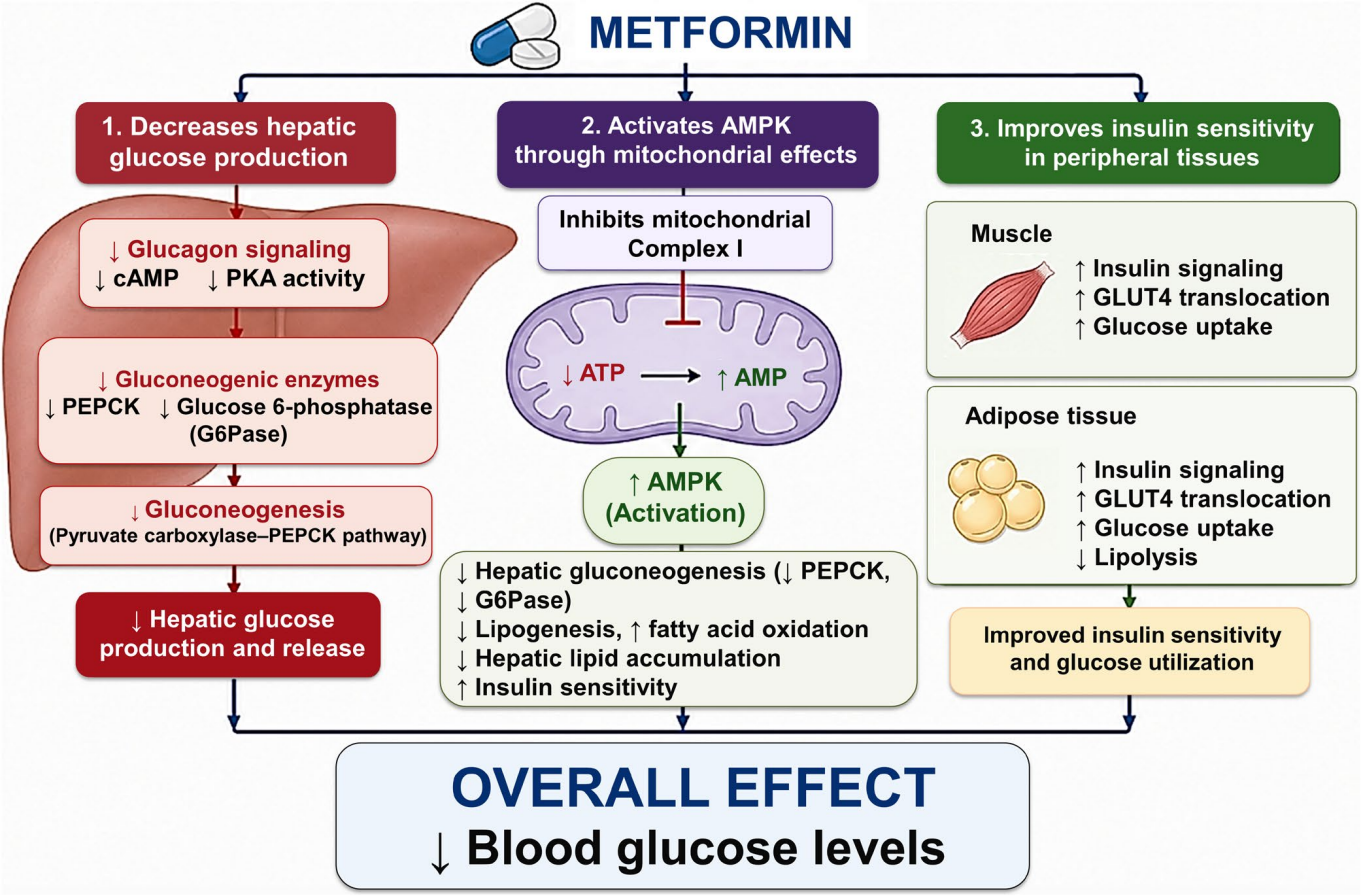
Mechanisms of action of metformin. Metformin lowers blood glucose through three major mechanisms. (1) Inhibition of hepatic glucose production: Metformin suppresses gluconeogenesis by downregulating phosphoenolpyruvate carboxykinase (PEPCK) and glucose‐6‐phosphatase (G6Pase), inhibiting glucagon‐mediated cAMP/PKA signalling, and blocking mitochondrial glycerophosphate dehydrogenase (mGPD), thereby reducing glucose production from lactate and glycerol. (2) AMPK activation: Inhibition of mitochondrial complex I decreases ATP and increases AMP, leading to activation of AMP‐activated protein kinase (AMPK), which suppresses gluconeogenesis and lipogenesis while promoting fatty acid oxidation. (3) Improved peripheral insulin sensitivity: Metformin enhances insulin signalling and GLUT4 translocation in skeletal muscle and adipose tissue, increasing glucose uptake and improving insulin sensitivity. Together, these actions reduce hepatic glucose output, enhance peripheral glucose utilization, and improve glycemic control.

### 4.2 Metformin and Transporter Polymorphisms

Metformin is required to enter cells and increase its concentration in the hepatocyte to inhibit hepatic glucose production, which involves membrane transport proteins encoded by solute carrier (SLC) genes. Metformin uptake is mediated by organic cation transporters (OCTs) and its efflux is regulated by multidrug and toxin extrusion (MATE) proteins, which regulate the distribution of metformin to the liver and intestine and its elimination in the kidney and bile [22, 23]. Polymorphisms in the SLC22 and SLC47 gene families may affect the expression or activity of the transporters, resulting in variations in the intracellular concentration of metformin, systemic exposure and clearance. Thus, inter‐individual differences in glycemic response and susceptibility to adverse effects, such as gastrointestinal intolerance and less frequently, lactic acidosis, are significant due to transporter variation. Therefore, transporter variation contributes to inter‐individual differences in glycemic response and susceptibility to adverse effects, especially gastrointestinal intolerance and less commonly, lactic acidosis [24]. The clinically relevant polymorphisms, functional implications and associations of the major transporter genes with metformin pharmacokinetics, efficacy and tolerability are summarized in Table 1.

**TABLE 1:** Metformin‐associated SNPs in OCT, MATE, and ATM transporter genes and their effects on metformin activity.

Gene	Transporter	SNP	Population/region	Main metformin effect	References
SLC22A1	OCT1	rs1867351	153 Han Chinese T2DM patients	T/T genotype associated with greater reductions in PPG and HbA1c after metformin	[25]
SLC22A1	OCT1	rs4709400	153 Han Chinese T2DM patients	G/G genotype associated with greater reductions in FPG and PPG after metformin	[25]
SLC22A1	OCT1	rs628031	Han Chinese	G/G genotype linked to greater FPG reduction	[25]
SLC22A1	OCT1	rs628031	Egyptian T2DM patients	A‐allele (AG/AA) associated with higher risk of gastrointestinal side effects	[26]
SLC22A1	OCT1	rs628031	Jordanian T2DM patients	A‐allele (AG/AA) associated with higher risk of side effects	[27]
SLC22A1	OCT1	rs2297374	Han Chinese T2DM patients	C/T genotype associated with greater reductions in FPG and HbA1c after metformin	[25]
SLC22A1	OCT1	rs622342	122 treatment‐naive South Indian T2DM patients	Variant significantly associated with metformin glycemic response	[28]
SLC22A1	OCT1	rs34130495	Danish T2DM patients (South Danish Diabetes Study)	Heterozygotes showed lower trough plasma metformin and attenuated HbA1c reduction (~1.1% less at 6 months)	[29]
SLC22A1	OCT1	rs72552763	Danish T2DM patients	Deletion carriers had lower plasma metformin and reduced HbA1c lowering	[29]
SLC22A1	OCT1	rs72552763	Egyptian T2DM patients	Associated with higher risk of metformin adverse effects	[26]
SLC22A1	OCT1	rs622342	Danish T2DM patients	Minor allele carriers tended to have lower trough plasma metformin, with no clear effect on HbA1c	[29]
SLC22A1	OCT1	rs622342	103 Mexican‐Mestizo T2DM patients	CC genotype associated with greater HbA1c reduction after metformin	[30]
SLC22A1	OCT1	rs628031	103 Mexican‐Mestizo T2DM patients	AA genotype associated with greater FPG reduction on metformin	[30]
SLC22A1	OCT1	rs594709	103 Mexican‐Mestizo T2DM patients	GG genotype associated with improved glycemic control (HbA1c lowering) with metformin	[30]
SLC22A1	OCT1	rs72552763	103 Mexican‐Mestizo T2DM patients	Carriers had lower plasmatic metformin concentration and attenuated HbA1c drop	[30]
SLC22A1	OCT1	rs1867351	212 Jordanian T2DM patients	T/T genotype associated with greater reductions in PPG and HbA1c after metformin	[27]
SLC22A1	OCT1	rs2282143	212 Jordanian T2DM patients	C/C genotype associated with improved HbA1c response to metformin	[27]
SLC22A1	OCT1	rs2297374	212 Jordanian T2DM patients	C/T genotype associated with greater reductions in FPG and HbA1c	[27]
SLC22A1	OCT1	rs461473	212 Jordanian T2DM patients	G/G genotype associated with stronger HbA1c reduction on metformin	[27]
SLC22A1	OCT1	rs4646272	212 Jordanian T2DM patients	Variant associated with trends toward altered metformin response (HbA1c lowering)	[27]
SLC22A1	OCT1	rs622342	212 Jordanian T2DM patients	CC genotype associated with HbA1c reduction; SNP linked to metformin response	[27]
SLC22A1	OCT1	rs683369	212 Jordanian T2DM patients	G/G genotype associated with improved glycemic control on metformin	[27]
SLC22A1	OCT1	rs622342	102 incident Caucasian metformin users	SNP significantly associated with glycemic outcomes during metformin therapy	[31]
SLC22A1	OCT1	rs1867351	103 healthy male Caucasians	C‐allele associated with increased renal clearance of metformin and reduced OCT1 expression; effect attenuated after adjustment	[32]
SLC22A1	OCT1	rs34059508	103 healthy male Caucasians	No significant association with metformin renal clearance	[32]
SLC22A1	OCT1	rs594709	267 T2DM patients and 182 healthy subjects (China)	Variant suggested to influence metformin therapeutic efficacy	[33]
SLC22A2	OCT2	rs316019	3234 US individuals with impaired glucose tolerance	No significant interaction with metformin therapy	[34]
SLC22A2	OCT2	rs316019	221 South Indian T2DM subjects	GG genotype associated with greater HbA1c reduction	[35]
SLC22A2	OCT2	rs2001844	26 healthy Korean volunteers	No significant difference in metformin pharmacokinetics compared with wild type	[36]
SLC22A2	OCT2	rs2001843	26 healthy Korean volunteers	No significant difference in metformin pharmacokinetics compared with wild type	[36]
SLC22A2	OCT2	rs316019	26 healthy Korean volunteers	Carriers had reduced renal clearance of metformin compared with wild type	[36]
SLC22A2	OCT2	rs10755577, rs17588242, rs17589858, rs2928035, rs3127573, rs316024, rs316025, rs316026, rs533452, rs662301	212 Jordanian T2DM patients	Tested variants not associated with metformin efficacy	[27]
SLC22A2	OCT2	rs10755577, rs2928035, rs316019, rs17588242, rs315996, rs316013, rs3127573, rs316024, rs316025, rs316026, rs662301, rs533452, rs17589858	103 healthy male Caucasians	Tested variants not associated with metformin renal clearance	[32]
SLC22A3	OCT3	rs12194182	212 Jordanian T2DM patients	Variant associated with better HbA1c levels during metformin therapy	[27]
SLC22A3	OCT3	rs3120137, rs3123634, rs12194182, rs2292334, rs2504927, rs2457576	103 healthy male Caucasians	No significant associations with metformin renal clearance	[32]
SLC22A3	OCT3	rs12194182, rs8187722	150 Iraqi T2DM patients	Variants not associated with clinical response to metformin	[37]
SLC22A3	OCT3	rs543159	200 Iranian T2DM patients	Variant carriers showed significant differences in HbA1c reduction after metformin	[38]
SLC22A3	OCT3	rs1317652	200 Iranian T2DM patients	Variant associated with variability in glycemic control; some genotypes linked to stronger metformin efficacy	[38]
SLC47A1	MATE1	rs2289669	7983 Caucasians aged ≥ 55 years	SNP associated with the glucose‐lowering effect of metformin	[39]
SLC47A1	MATE1	rs2289669	267 T2DM patients and 182 healthy subjects (China)	Variant may influence metformin therapeutic effectiveness	[33]
SLC47A1	MATE1	rs2289669	South Indian T2DM patients	No significant association with metformin response	[40]
SLC47A2	MATE2	rs12943590	Iraqi T2DM patients	Variant not associated with clinical response to metformin	[37]
SLC47A2	MATE2	rs12943590	221 South Indian T2DM patients	GA genotype associated with more pronounced HbA1c reduction on metformin	[35]
SLC47A2	MATE2	rs12943590	105 South Indian T2DM patients on metformin monotherapy	No significant association with glycemic response to metformin	[40]
ATM	ATM	rs11212617	274 incident metformin users in China	Variant associated with variability in metformin response	[41]
ATM	ATM	rs11212617	1365 Europeans with T2DM	Variant associated with variability in metformin efficacy	[42]

*Note:* Associations are based on individual studies and do not support routine genotype‐guided metformin therapy.

Abbreviations: ATM, ataxia‐telangiectasia mutated; FPG, fasting plasma glucose; HbA1c, glycated haemoglobin; MATE1/2, multidrug and toxin extrusion proteins 1/2; OCT1/2/3, organic cation transporters 1/2/3; PPG, postprandial glucose; SNP, single nucleotide polymorphism; T2DM, type 2 diabetes mellitus.

#### 4.2.1 SLC22A1 Variants and Metformin Activity

SLC22A1 is a gene that encodes OCT1, the main hepatic uptake transporter that facilitates the entry of metformin into hepatocytes, where it inhibits hepatic gluconeogenesis [43, 44]. SLC22A1 functional variants affect the activity of OCT1 and intracellular accumulation of metformin. Reduced‐function alleles are linked to decreased glycemic efficacy and increased gastrointestinal drug exposure and gastrointestinal intolerance, while normal‐function alleles are linked to better therapeutic response [45, 46]. Therefore, SLC22A1 is one of the most consistent genetic markers of metformin response, with variants consistently associated with metformin pharmacokinetics, HbA1c reduction and treatment tolerability [28, 29, 47].

#### 4.2.2 SLC22A2 Variants and Metformin Activity

The major renal uptake transporter for metformin uptake into proximal tubular cells prior to urinary secretion is encoded by SLC22A2 [48]. The functional variants do not significantly alter the glucose‐lowering activity but rather renal clearance. Reduced‐function alleles lead to lower renal clearance and higher systemic exposure, while normal‐function alleles lead to higher renal clearance and lower systemic exposure [36]. The association of SLC22A2 polymorphisms with altered metformin pharmacokinetics (particularly renal clearance and steady‐state plasma concentrations) is consistent but generally weaker than that of SLC22A1 [27, 34, 35, 36].

#### 4.2.3 SLC22A3 Variants and Metformin Activity

SLC22A3 is the gene for OCT3, a poly‐specific transporter that is expressed in the liver, skeletal muscle, heart, and gastrointestinal tract and is involved in the tissue distribution of metformin. Functional variants can affect intracellular exposure to drugs and the pharmacodynamic response [49]. While less comprehensive than for OCT1 and OCT2, there are some studies that link SLC22A3 polymorphisms with variations in metformin pharmacokinetics and glycemic effects, indicating a moderate role in inter‐individual variability in treatment outcomes [27, 32, 37, 38].

#### 4.2.4 SLC47A1 Variants and Metformin Activity

SLC47A1 encodes MATE1, an apical efflux transporter that mediates metformin excretion into bile and urine, thereby regulating intracellular drug concentrations and systemic clearance [50]. Reduced‐function variants increase intracellular and plasma metformin concentrations, potentially enhancing glycemic efficacy but also increasing concentration‐dependent adverse effects, whereas higher‐activity alleles promote faster elimination [51]. Consistent with these mechanisms, SLC47A1 polymorphisms have been associated with differences in metformin pharmacokinetics, insulin resistance and glycemic outcomes [33, 39, 40].

#### 4.2.5 SLC47A2 Variants and Metformin Activity

SLC47A2 encodes MATE2, a renal apical efflux transporter that acts with MATE1 to mediate urinary metformin secretion [52]. Functional variants primarily influence renal clearance, with reduced‐function alleles increasing systemic exposure and modestly enhancing glycemic response while raising the risk of concentration‐dependent adverse effects [53]. Overall, evidence indicates that SLC47A2 polymorphisms contribute modestly to variability in metformin pharmacokinetics and treatment response [35, 37, 40].

#### 4.2.6 Ataxia Telangiectasia Mutated (ATM) Variants and Metformin Activity

Unlike transporter genes, the ATM gene influences metformin response through intracellular signalling rather than drug disposition. ATM encodes a serine/threonine kinase functionally linked to AMP‐activated protein kinase (AMPK), a central mediator of metformin‐induced suppression of hepatic gluconeogenesis [54]. Variants that alter ATM‐mediated signalling may modify hepatocyte sensitivity to metformin and contribute to differences in glycemic response [55]. Consistent with this mechanism, common ATM polymorphisms have been associated with variability in HbA1c reduction following metformin therapy [41, 42].

## 5 SGLT2 Inhibitors

Despite the availability of numerous glucose‐lowering therapies, many conventional antidiabetic drugs remain insufficient to prevent the long‐term complications associated with diabetes. To overcome these limitations, the U.S. Food and Drug Administration (FDA) approved a newer class of anti‐hyperglycemic agents known as sodium‐glucose cotransporter‐2 (SGLT2) inhibitors. This class includes canagliflozin, dapagliflozin, empagliflozin, ertugliflozin and bexagliflozin. These agents lower blood glucose by selectively inhibiting SGLT2 in the proximal convoluted tubules of the kidney, reducing renal glucose reabsorption and increasing urinary glucose excretion. By targeting renal glucose handling rather than insulin secretion or action, SGLT2 inhibitors introduced a distinct therapeutic approach for improving glycemic control [56].

The development of SGLT2 inhibitors originated from phlorizin, a naturally occurring dihydrochalcone isolated from the bark of apple trees [57]. As early as the 1930s, studies showed that phlorizin blocked renal glucose reabsorption in the proximal tubules, leading to increased urinary glucose excretion [58]. Subsequent experiments in diabetic animal models demonstrated that phlorizin effectively lowered plasma glucose concentrations while improving insulin sensitivity and overall glucose homeostasis. Although its poor pharmacokinetic properties and lack of selectivity limited its clinical application, phlorizin provided the structural and mechanistic foundation for the development of the highly selective SGLT2 inhibitors used in clinical practice today [59, 60].

### 5.1 Mechanism of Action

The two sodium‐glucose cotransporters identified in humans are SGLT1 and SGLT2. SGLT2 is expressed predominantly in the kidneys, whereas SGLT1 is found in the small intestine, heart, skeletal muscle and kidneys [49, 61, 62]. In the kidneys, these transporters mediate sodium and glucose reabsorption in the proximal tubules of the nephrons.

Under normal physiological conditions, SGLT2 reabsorbs approximately 90%–97% of filtered glucose in the proximal tubule, while SGLT1 reabsorbs the remaining 3%–10% in the S3 segment [63]. Glucose exits tubular epithelial cells through GLUT2 following SGLT2‐mediated uptake and through GLUT1 following SGLT1‐mediated uptake. The Na^+^/K^+^‐ATPase maintains the sodium gradient that drives glucose transport, while the complementary kinetics of high‐capacity, low‐affinity SGLT2 and low‐capacity, high‐affinity SGLT1 ensure efficient glucose reabsorption across varying plasma glucose concentrations. This coordinated process maintains glucose homeostasis and minimizes urinary glucose loss under normal conditions [56].

In persistent hyperglycemia, renal SGLT2 expression is upregulated, resulting in increased proximal tubular glucose reabsorption and contributing to sustained hyperglycemia. Although SGLT2 inhibitors exert only modest glucose‐lowering effects, substantial clinical trial evidence demonstrates significant cardiovascular and renoprotective benefits, establishing them as a cornerstone of T2DM management [64, 65] (Figure 3).

**FIGURE 3:**
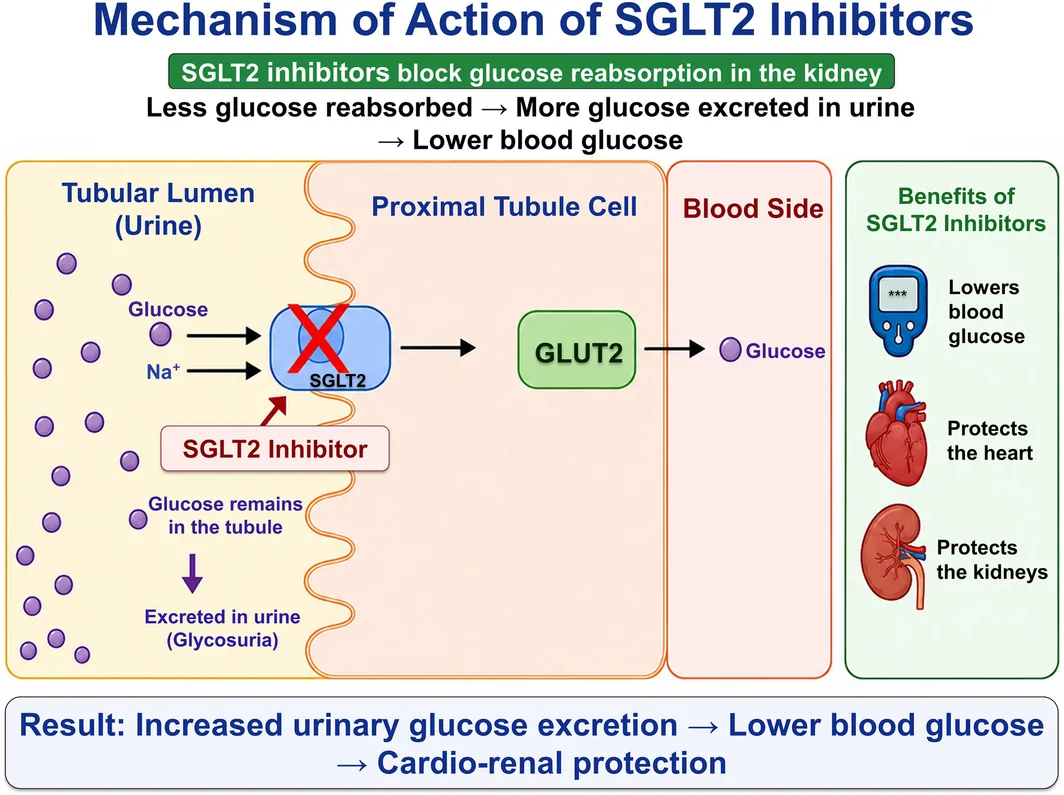
Mechanism of action of SGLT2 inhibitors. Sodium‐glucose cotransporter 2 (SGLT2), located in the proximal tubule of the nephron, is responsible for the reabsorption of the majority of filtered glucose from the tubular lumen back into the bloodstream. SGLT2 inhibitors block this transporter, thereby reducing renal glucose reabsorption and increasing urinary glucose excretion (glycosuria). The resulting loss of glucose in urine lowers blood glucose levels independently of insulin action. Beyond glycemic control, SGLT2 inhibitors provide additional cardiovascular and renal benefits, including protection against heart failure and slowing the progression of chronic kidney disease.

### 5.2 SGLT2 Inhibitors and Transporter/Metabolizing Enzyme Polymorphisms

The pharmacokinetics and therapeutic response of SGLT2 inhibitors are influenced by both drug‐metabolizing enzymes and transport‐related proteins. For canagliflozin, dapagliflozin and ertugliflozin, glucuronidation by UGT1A9 and UGT2B4 is the primary metabolic pathway, converting the parent drugs into inactive glucuronide metabolites in the liver and kidneys. In addition, genetic variation in the drug target, SLC5A2 and glucose transporter genes (GLUT1, GLUT2, GLUT4 and GLUT9) may modify renal and systemic glucose handling, thereby influencing metabolic traits and the pharmacodynamic response to SGLT2 inhibition [66]. These polymorphisms can contribute to inter‐individual variability in drug exposure, glycemic control and cardiorenal outcomes; however, their effects are generally modest and currently insufficient to support routine genotype‐guided dosing [67]. The following subsections summarize the major human pharmacogenetic findings for UGT1A9, UGT2B4, PNPLA3, SLC5A2, and selected GLUT genes, with detailed variant information and associated clinical phenotypes presented in Table 2.

**TABLE 2:** Genetic polymorphisms associated with the pharmacokinetics, pharmacodynamics, and clinical response of SGLT2 inhibitors.

Gene	Transporter/protein	SNP (genotype, if specified)	Population/region	Main SGLT2 inhibitors effect	References
UGT1A9	UDP‐glucuronosyltransferase 1A9 (drug‐metabolizing enzyme)	rs72551330 T>C (UGT1A9*3)	Non‐diabetic participants in Phase I canagliflozin studies (*n* = 131)	UGT1A9*3 carriers had ~1.45‐fold higher canagliflozin AUC, indicating increased systemic exposure	[68]
UGT1A9	UDP‐glucuronosyltransferase 1A9	rs72551330 T>C (UGT1A9*3)	Pooled Phase I‐III canagliflozin trials in diabetic and non‐diabetic participants (*n* = 1616)	UGT1A9*3 carriers showed ~1.26‐fold higher dose‐normalized canagliflozin AUC; the increase was not clinically meaningful and did not warrant dose adjustment	[69]
UGT1A9	UDP‐glucuronosyltransferase 1A9	rs2741049, rs2011404, rs6759892, rs7577677, rs4148323, UGT1A9*2, UGT1A9*3	Phase II dapagliflozin‐treated T2DM patients (*n* = 187)	None of the UGT1A9 polymorphisms had a clinically relevant effect on dapagliflozin apparent oral clearance (CL/F) compared with the wild‐type genotype	[70]
UGT1A9	UDP‐glucuronosyltransferase 1A9	UGT1A9–2152 (C>T), UGT1A9*3, UGT1A9*1b	Pooled analysis of 25 Phase I ertugliflozin studies in healthy subjects (0.5–300 mg)	UGT1A9*3 heterozygous carriers had the largest increase in ertugliflozin AUC, but all UGT1A9 variant effects on exposure were within ±10% of wild type and not clinically meaningful; no dose adjustment was required	[71]
UGT1A9	UDP‐glucuronosyltransferase 1A9	rs12988520	Chinese T2DM patients treated with dapagliflozin	AC/CC carriers showed higher fasting blood glucose after dapagliflozin, but the association was not significant after multiple‐testing correction; no robust association with treatment response	[72]
UGT1A9	UDP‐glucuronosyltransferase 1A9	rs12471030	Chinese T2DM patients treated with dapagliflozin	CT/TT carriers had higher fasting blood glucose after dapagliflozin, but this association lost significance after Bonferroni adjustment; no confirmed impact on drug response	[72]
UGT2B4	UDP‐glucuronosyltransferase 2B4	rs1080755	Egyptian heart failure patients on dapagliflozin	Variant associated with improved KCCQ‐12 quality‐of‐life score in patients with preserved ejection fraction receiving dapagliflozin, without a significant effect on the primary clinical outcome	[73]
PNPLA3	Patatin‐like phospholipase domain‐containing protein 3	rs738409	T2DM and NAFLD subjects: 20 on dapagliflozin alone and 20 on dapagliflozin plus omega‐3 carboxylic acids	CG/GG genotype associated with smaller liver PDFF reduction under dapagliflozin monotherapy but greater reduction when dapagliflozin was combined with omega‐3 carboxylic acids, indicating genotype‐dependent effects on liver fat response	[74]
SLC2A1	GLUT1 (glucose transporter 1)	rs1385129	Egyptian heart failure patients on dapagliflozin	No significant association between rs1385129 and dapagliflozin efficacy or cardiovascular outcomes	[73]
SLC2A2	GLUT2 (glucose transporter 2)	rs5398	Chinese T2DM patients treated with dapagliflozin	No significant association with fasting blood glucose or HbA1c response after dapagliflozin treatment	[72]
SLC2A2	GLUT2 (glucose transporter 2)	rs2602381	Chinese T2DM patients treated with dapagliflozin	TC/CC carriers had higher fasting blood glucose after treatment, but the association was not significant after Bonferroni correction; no robust effect on dapagliflozin response	[72]
SLC2A4	GLUT4 (glucose transporter 4)	rs5418, rs5435, rs16956647	Chinese T2DM patients treated with dapagliflozin	None of the tested GLUT4 variants showed significant associations with dapagliflozin‐induced glucose lowering after multiple‐testing correction	[72]
SLC2A9	GLUT9 (urate and hexose transporter)	rs734553	Chinese male Han participants: 167 T2DM only, 198 hyperuricemia only, 115 T2DM + hyperuricemia, 128 healthy controls	C allele associated with higher risk of hyperuricemia and with T2DM when co‐existing with hyperuricemia; carriers had elevated serum uric acid and worse metabolic measures, with no CC homozygotes observed and no association with isolated T2DM	[75]
SLC5A2	SGLT2 transporter (drug target)	rs9934336, rs3813007, rs3813008, rs3116150	German cohorts: Sorb (106 with and 907 without T2DM) and Berlin Potsdam study (359 with and 1683 without T2DM)	No significant association of these polymorphisms with T2DM risk; in non‐diabetic subjects, rs9934336 AA genotype associated with lower 30‐min glucose and reduced 120‐min insulin during OGTT, suggesting effects on metabolic traits rather than drug response	[76]
SLC5A2	SGLT2 transporter (drug target)	rs9924771, rs3116150, rs3813008, rs9934336	375 individuals at increased risk of T2DM	No significant association between SLC5A2 polymorphisms and fasting or OGTT‐derived plasma glucagon concentrations, indicating no clear effect on glucagon‐related metabolic traits	[77]
SLC5A2	SGLT2 transporter (drug target)	rs9934336, rs3813008, rs3116150	1684 individuals undergoing coronary angiography, including 400 with T2DM; meta‐analysis of three studies	rs9934336 associated with reduced HbA1c and lower T2DM risk, with no association to coronary artery disease or cardiovascular events; meta‐analysis confirmed reduced T2DM risk linked to rs9934336	[78]
SLC5A2	SGLT2 transporter (drug target)	rs9934336	181 Slovenian T2DM patients	rs9934336 A allele associated with higher fasting glucose, higher HbA1c, and increased risk of diabetic retinopathy; no association with other diabetic microvascular or macrovascular complications	[79]
SLC5A2	SGLT2 transporter (drug target)	rs3116149, rs9934336, rs3813008, rs11646054, rs3116650, rs9924771	2229 participants from the Tübingen Family Study (1558 glucose‐tolerant, 671 prediabetic); 603 T2DM on empagliflozin and 305 on placebo	No significant associations between these SLC5A2 variants and metabolic traits or clinical response to empagliflozin treatment	[80]

*Note:* Associations are based on individual studies and do not support routine genotype‐guided SGLT2 inhibitor therapy.

Abbreviations: AUC, area under the concentration‐time curve; CL/F, apparent oral clearance; GLUT1/2/4/9, glucose transporters 1/2/4/9; HbA1c, glycated haemoglobin; KCCQ‐12, Kansas City Cardiomyopathy Questionnaire‐12; NAFLD, non‐alcoholic fatty liver disease; OGTT, oral glucose tolerance test; PDFF, proton density fat fraction; SGLT2, sodium‐glucose cotransporter 2; SNP, single nucleotide polymorphism; T2DM, type 2 diabetes mellitus; UGT1A9/UGT2B4, uridine diphosphate‐glucuronosyltransferases 1A9/2B4.

#### 5.2.1 UGT1A9 Variants and SGLT2 Inhibitors Activity

UGT1A9 encodes a major uridine diphosphate‐glucuronosyltransferase responsible for the glucuronidation of several SGLT2 inhibitors, including canagliflozin, dapagliflozin and ertugliflozin, thereby contributing to their hepatic and renal biotransformation and systemic clearance [81]. Functional variants, including UGT1A9*3 (p.Met33Thr), reduce enzyme activity and modestly increase drug exposure by slowing glucuronidation. Pharmacokinetic studies have shown higher area under the curve (AUC) values for canagliflozin and ertugliflozin in carriers of reduced‐function alleles; however, these changes have not translated into clinically meaningful differences in glycemic efficacy or dose requirements [67]. Thus, UGT1A9 primarily influences pharmacokinetic variability rather than therapeutic response.

#### 5.2.2 UGT2B4 Variants and SGLT2 Inhibitors Activity

UGT2B4, together with UGT1A9, mediates the glucuronidation of several SGLT2 inhibitors, particularly canagliflozin, facilitating their conversion to inactive metabolites and systemic clearance [82]. Reduced‐function polymorphisms may decrease glucuronidation efficiency and modestly increase plasma drug exposure. Although carriers of these variants exhibit higher canagliflozin concentrations, the pharmacokinetic differences are generally small and have not resulted in clinically significant changes in glucose‐lowering efficacy or dose requirements [73, 82, 83]. Current evidence therefore supports a predominantly pharmacokinetic, rather than pharmacodynamic, role for UGT2B4 variation.

#### 5.2.3 PNPLA3 Variants and SGLT2 Inhibitors Activity

PNPLA3 encodes patatin‐like phospholipase domain‐containing protein 3, a key regulator of hepatic triglyceride metabolism and a major genetic determinant of non‐alcoholic fatty liver disease (NAFLD). The rs738409 (I148M) variant is associated with increased hepatic steatosis and fibrosis in patients with type 2 diabetes and NAFLD [84]. Although PNPLA3 does not directly metabolize SGLT2 inhibitors, it may influence hepatic responses to treatment. In a randomized trial, carriers of the I148M risk allele showed smaller reductions in liver proton density fat fraction (PDFF) with dapagliflozin alone but greater improvements when dapagliflozin was combined with omega‐3 carboxylic acids, suggesting genotype‐dependent modulation of hepatic outcomes rather than glycemic efficacy [74].

#### 5.2.4 SLC5A2 Variants and SGLT2 Inhibitors Activity

SLC5A2 encodes SGLT2, the principal renal glucose transporter and direct molecular target of all approved SGLT2 inhibitors. Loss‐of‐function or hypomorphic variants cause familial renal glucosuria by lowering the renal glucose threshold, thereby partially mimicking pharmacological SGLT2 inhibition [85]. Common polymorphisms, including rs9934336 and rs3813008, have been associated with variation in glucose homeostasis, HbA1c, fasting glucose and type 2 diabetes risk [76, 78]. More recent studies suggest that rs3813008 may also influence cardiorenal outcomes and dapagliflozin response, possibly reflecting pre‐existing ‘natural SGLT2 inhibition’ that limits additional therapeutic benefit [73]. Nevertheless, genotype‐guided therapy remains investigational.

#### 5.2.5 SLC2A1 Variants and SGLT2 Inhibitors Activity

SLC2A1 encodes GLUT1, a ubiquitously expressed facilitative glucose transporter that maintains basal glucose uptake and contributes to systemic glucose homeostasis. Consequently, genetic variation may indirectly influence the metabolic environment in which SGLT2 inhibitors act [86]. However, evidence linking SLC2A1 polymorphisms to treatment response remains limited. In an Egyptian heart failure cohort receiving dapagliflozin, the common rs1385129 variant showed no significant association with glycemic or cardiovascular outcomes, suggesting that GLUT1 has, at most, a modest influence on SGLT2 inhibitor efficacy [73].

#### 5.2.6 SLC2A2 Variants and SGLT2 Inhibitors Activity

SLC2A2 encodes GLUT2, a low‐affinity, high‐capacity glucose transporter expressed in the liver, kidney, pancreatic β‐cells and intestine, where it regulates glucose transport and sensing [87]. Although SLC2A2 polymorphisms influence fasting glucose, insulin secretion and long‐term glycemic control, studies evaluating their impact on dapagliflozin response have not demonstrated consistent or clinically meaningful effects after correction for multiple testing. Current evidence therefore suggests only a limited indirect contribution of GLUT2 variation to SGLT2 inhibitor efficacy [72].

#### 5.2.7 SLC2A4 Variants and SGLT2 Inhibitors Activity

SLC2A4 encodes GLUT4, the insulin‐responsive glucose transporter responsible for glucose uptake in skeletal muscle and adipose tissue and a major determinant of insulin sensitivity [88]. Although polymorphisms in SLC2A4 have been associated with variation in insulin sensitivity and glycemic traits, pharmacogenetic studies have not identified robust or clinically meaningful effects on dapagliflozin‐induced glucose lowering after adjustment for multiple comparisons. Thus, current evidence does not support GLUT4 as a major determinant of SGLT2 inhibitor response [72].

#### 5.2.8 SLC2A9 Variants and SGLT2 Inhibitors Activity

SLC2A9 encodes GLUT9, a facilitative transporter involved in both glucose and urate transport that plays a central role in renal urate reabsorption [89]. Because SGLT2 inhibitors lower serum uric acid by promoting uricosuria, GLUT9 may influence this secondary therapeutic effect. Although SLC2A9 polymorphisms have been associated with serum uric acid concentrations, hyperuricemia risk and related metabolic traits, current evidence does not support a substantial effect on the primary glycemic or cardiorenal benefits of SGLT2 inhibitors. Their clinical relevance therefore appears largely confined to urate‐related outcomes [90].

## 6 Comparison Between Metformin and SGLT2 Inhibitors

Metformin and SGLT2 inhibitors represent two cornerstone, yet mechanistically distinct, therapies in type 2 diabetes management. Metformin, a biguanide, primarily lowers glucose by suppressing hepatic gluconeogenesis and improving insulin sensitivity, whereas SGLT2 inhibitors reduce glycemia by promoting renal glucose loss via blockade of the SGLT2 transporter in the proximal tubule. These differences in site and mode of action translate into divergent profiles for insulin dependence, weight change, cardiovascular and renal protection, and adverse events, making them complementary rather than interchangeable options in clinical practice [91, 92].

From a pharmacogenetic perspective, metformin response is strongly shaped by variation in transporters such as OCT1, OCT2 and MATE1/2, and in signalling genes like ATM, leading to substantial inter‐individual differences in exposure and HbA1c reduction [93]. In contrast, pharmacogenomic research on SGLT2 inhibitors is more recent and has focused on UGT enzymes and SLC5A2 (SGLT2) variants, with emerging but generally modest effects on glycemic and cardiorenal outcomes [94]. These clinical, mechanistic and genetic contrasts are summarized in Table 3, which outlines the key features distinguishing metformin from SGLT2 inhibitors and highlights their respective implications for gene‐guided therapy in T2DM.

**TABLE 3:** Comparative clinical, mechanistic, and pharmacogenetic features of metformin and SGLT2 inhibitors in type 2 diabetes mellitus.

Feature	Metformin	SGLT2 inhibitors	References
Drug class	Biguanide; first‐line oral antihyperglycemic agent	Sodium‐glucose cotransporter‐2 inhibitors (‘gliflozins’)	[9]
Primary site of action	Liver (decreased hepatic gluconeogenesis) and intestine (reduced glucose absorption)	Kidney proximal tubule (reduced glucose reabsorption via SGLT2 inhibition)	[95]
Mechanism of action	Activates AMPK, leading to reduced hepatic glucose production and improved insulin sensitivity	Inhibits SGLT2 (SLC5A2), increasing urinary glucose excretion	[96]
Insulin dependence	Insulin‐independent glucose‐lowering, with secondary improvement in insulin sensitivity	Completely insulin‐independent mechanism of glucose lowering	[97]
Metabolism	Not metabolized; excreted unchanged in urine	Primarily metabolized by hepatic glucuronidation via UGT1A9; minimal involvement of CYP enzymes	[10]
Elimination	Renal excretion via OCT2 and MATE transporters	Elimination through increased renal glucose excretion plus hepatic metabolism	[50, 82]
Key transporters/targets	OCT1 (SLC22A1), OCT2 (SLC22A2), MATE1/2 (SLC47A1/2)	SGLT2 (SLC5A2) in the proximal renal tubule	[11]
Genetic influence	Well‐described pharmacogenetic variability in response and clearance driven by OCT/MATE variants	Emerging pharmacogenomic evidence for SGLT2‐related and metabolic gene variants modulating response	[46]
Weight effect	Generally weight‐neutral, with possible mild weight loss	Consistently associated with clinically meaningful weight reduction	[97]
Hypoglycemia risk	Low risk, except when combined with insulin or sulfonylureas	Very low hypoglycemia risk due to insulin‐independent action	[97]
Cardiovascular benefit	Modest or ‘legacy’ cardiovascular benefit in T2DM	Robust reductions in heart failure hospitalization and cardiovascular mortality	[97]
Renal benefit	Mild nephroprotective effects	Strong renoprotective effects, including slowing chronic kidney disease progression	[95]
Major adverse effects	Gastrointestinal intolerance, rare lactic acidosis	Genital mycotic infections, volume depletion, and rare euglycemic ketoacidosis	[98]
Drug interaction profile	Transporter‐based interactions; inhibition of OCT/MATE can increase metformin exposure	Additive diuretic effect with other diuretics; overall minimal CYP‐mediated drug interactions	[99, 100]

*Note:* The comparison summarizes the general pharmacological and pharmacogenetic characteristics of metformin and SGLT2 inhibitors.

Abbreviations: AMPK, AMP‐activated protein kinase; CYP, cytochrome P450; MATE1/2, multidrug and toxin extrusion proteins 1/2; OCT1/2, organic cation transporters 1/2; SGLT2, sodium‐glucose cotransporter 2; T2DM, type 2 diabetes mellitus; UGT1A9, uridine diphosphate‐glucuronosyltransferase 1A9.

## 7 Discussion

The present review highlights the growing body of evidence demonstrating that genetic variation is an important determinant of the marked inter‐individual variability observed in the therapeutic response to metformin and SGLT2 inhibitors in patients with T2DM. Although both drug classes have consistently demonstrated robust glycemic efficacy and favourable safety profiles in large randomized clinical trials, substantial heterogeneity persists in treatment response, long‐term glycemic durability, and susceptibility to adverse drug reactions among patients receiving comparable therapeutic regimens [3, 4]. The accumulated pharmacogenetic evidence suggests that inherited variants affecting drug transporters, metabolizing enzymes and intracellular signalling pathways contribute significantly to this variability, thereby providing a biological basis for the development of precision medicine approaches in T2DM [6, 93]. Nevertheless, the strength of evidence differs considerably between drug classes, and important challenges remain before pharmacogenetic information can be translated into routine clinical practice.

A key observation emerging from this review is the disparity in the maturity of pharmacogenetic evidence between metformin and SGLT2 inhibitors. Among currently available oral glucose‐lowering agents, metformin possesses the most comprehensive pharmacogenetic profile, with transporter genes consistently identified as major determinants of drug disposition and therapeutic response. In particular, SLC22A1 (OCT1), SLC22A2 (OCT2), SLC22A3 (OCT3), SLC47A1 (MATE1) and SLC47A2 (MATE2) regulate hepatic uptake, intracellular accumulation, and renal elimination of metformin, thereby directly influencing systemic drug exposure and glucose‐lowering efficacy [22, 24, 43]. Functional variants within these transporters have been associated with differences in HbA1c reduction, fasting plasma glucose, and gastrointestinal intolerance, highlighting the central role of pharmacokinetic mechanisms in determining therapeutic outcomes [28, 45, 46]. Beyond transporter‐mediated effects, variants in the ATM gene further demonstrate that pharmacodynamic mechanisms also contribute to treatment variability through modulation of AMPK activation and hepatic gluconeogenesis [41, 55]. Collectively, these findings indicate that metformin response is governed by a complex interaction between pharmacokinetic and pharmacodynamic pathways rather than by alterations in a single molecular target. The overall strength of evidence supporting the principal pharmacogenetic markers discussed in this review is summarized in Table 4.

**TABLE 4:** Overall level of evidence and clinical applicability of pharmacogenetic markers associated with metformin and SGLT2 inhibitor response.

Drug class	Gene	Primary role	Main clinical association	Reproducibility of evidence	Overall evidence level^a^	Current clinical applicability
Metformin	SLC22A1 (OCT1)	Hepatic uptake transporter	HbA1c reduction, FPG, PPG, gastrointestinal intolerance, metformin exposure	High (multiple independent populations)	High	Most promising biomarker; routine testing not yet recommended
	SLC22A2 (OCT2)	Renal uptake transporter	Renal clearance, plasma exposure, HbA1c	Moderate	Moderate	Investigational
	SLC22A3 (OCT3)	Tissue uptake transporter	Glycemic response	Moderate	Moderate	Investigational
	SLC47A1 (MATE1)	Hepatic/renal efflux transporter	HbA1c response, metformin disposition	Moderate	Moderate	Investigational
	SLC47A2 (MATE2)	Renal efflux transporter	HbA1c response, renal clearance	Moderate but inconsistent	Low‐moderate	Investigational
	ATM	AMPK signalling	Glycemic response	Moderate	Moderate	Investigational
SGLT2 inhibitors	UGT1A9	Drug metabolism	Increased AUC; minimal effect on efficacy	High consistency for PK; limited PD relevance	Moderate	No dose adjustment recommended
	UGT2B4	Drug metabolism	Small increase in exposure	Moderate	Low‐moderate	No clinical implementation
	SLC5A2	Drug target	Glycemic traits, possible cardiorenal response	Moderate but inconsistent	Moderate	Promising; requires validation
	PNPLA3	Hepatic lipid metabolism	Liver fat reduction in NAFLD	Limited	Low	Exploratory
	SLC2A1 (GLUT1)	Basal glucose transport	No consistent association	Low	Low	None
	SLC2A2 (GLUT2)	Glucose sensing/transport	No robust association	Low	Low	None
	SLC2A4 (GLUT4)	Peripheral glucose uptake	No robust association	Low	Low	None
	SLC2A9 (GLUT9)	Uric acid transport	Uricosuric response	Limited	Low	Exploratory

^a^ Evidence level reflects the overall consistency of published human pharmacogenetic studies, replication across independent populations, biological plausibility, and demonstrated clinical relevance, rather than the statistical significance of individual studies.

Despite the biological plausibility of these associations, their reproducibility across different populations remains inconsistent, representing one of the major barriers to clinical implementation. Several SLC22A1 polymorphisms have demonstrated significant associations with metformin response in Chinese, Jordanian, South Indian and Mexican populations, whereas similar associations were not replicated in other ethnic groups [27, 28, 30]. Likewise, conflicting evidence has been reported for variants in SLC47A1 and SLC47A2, with some studies reporting improved glycemic outcomes while others failed to identify statistically significant associations [35, 39, 40]. These discrepancies likely reflect differences in study design, statistical power, ethnic diversity, allele frequencies, treatment duration, baseline glycemic status, renal function, concomitant medications and environmental exposures. Importantly, they also underscore the limitations of the traditional candidate‐gene approach, suggesting that therapeutic response cannot be adequately explained by individual polymorphisms in isolation. Instead, these observations support the concept that multiple genetic variants with modest individual effects collectively determine clinical response.

Compared with metformin, pharmacogenomic evidence for SGLT2 inhibitors remains relatively sparse and less conclusive. Current investigations have focused primarily on polymorphisms in UGT1A9, UGT2B4, SLC5A2, and selected glucose transporter genes. Although reduced‐function variants in UGT1A9 and UGT2B4 have been shown to modestly increase systemic drug exposure by reducing glucuronidation, these pharmacokinetic alterations have generally not translated into clinically meaningful improvements in glycemic control or justified genotype‐guided dose adjustment [68, 69, 71, 83]. Similarly, common SLC5A2 polymorphisms have been associated with glucose homeostasis and diabetes susceptibility in certain populations; however, evidence linking these variants to therapeutic efficacy remains inconsistent and insufficient for clinical application [76, 78, 80]. Taken together, these findings suggest that, unlike metformin, currently identified pharmacogenetic markers for SGLT2 inhibitors exert relatively modest clinical effects and have yet to demonstrate consistent predictive utility.

An emerging theme identified in this review is that the pharmacogenetic relevance of SGLT2 inhibitors may extend beyond glycemic control. Variants in PNPLA3 have been associated with differential reductions in hepatic steatosis among patients with T2DM and non‐alcoholic fatty liver disease receiving dapagliflozin, whereas polymorphisms in SLC2A9 may influence uric acid metabolism and hyperuricemia‐related outcomes [74, 75]. Furthermore, recent evidence suggests that SLC5A2 variants may contribute to inter‐individual differences in cardiovascular and renal outcomes following SGLT2 inhibitor therapy, although these observations remain preliminary and require confirmation in adequately powered prospective studies [73, 101]. These findings broaden the scope of pharmacogenomic research beyond conventional glycemic endpoints and raise the possibility that host genetics may also influence the pleiotropic cardiorenal and metabolic benefits increasingly recognized for this therapeutic class.

Another important insight emerging from the available literature is that drug response in T2DM is inherently polygenic and multifactorial. Rather than being determined by a single genetic variant, therapeutic outcomes reflect the cumulative influence of numerous polymorphisms affecting drug transport, metabolism, intracellular signalling, insulin sensitivity, β‐cell function, glucose homeostasis and renal glucose handling [14, 15]. Moreover, genetic susceptibility operates within a complex clinical and environmental context in which factors such as age, sex, obesity, renal function, dietary habits, physical activity, concomitant medications, and gut microbiota substantially modify treatment response [5, 9]. Consequently, reliance on single‐gene pharmacogenetic testing is unlikely to provide sufficient predictive accuracy for routine clinical decision‐making. Future predictive models will likely require integration of multiple genetic variants with clinical characteristics and other biological data to improve individualized treatment prediction (Figure 4).

**FIGURE 4:**
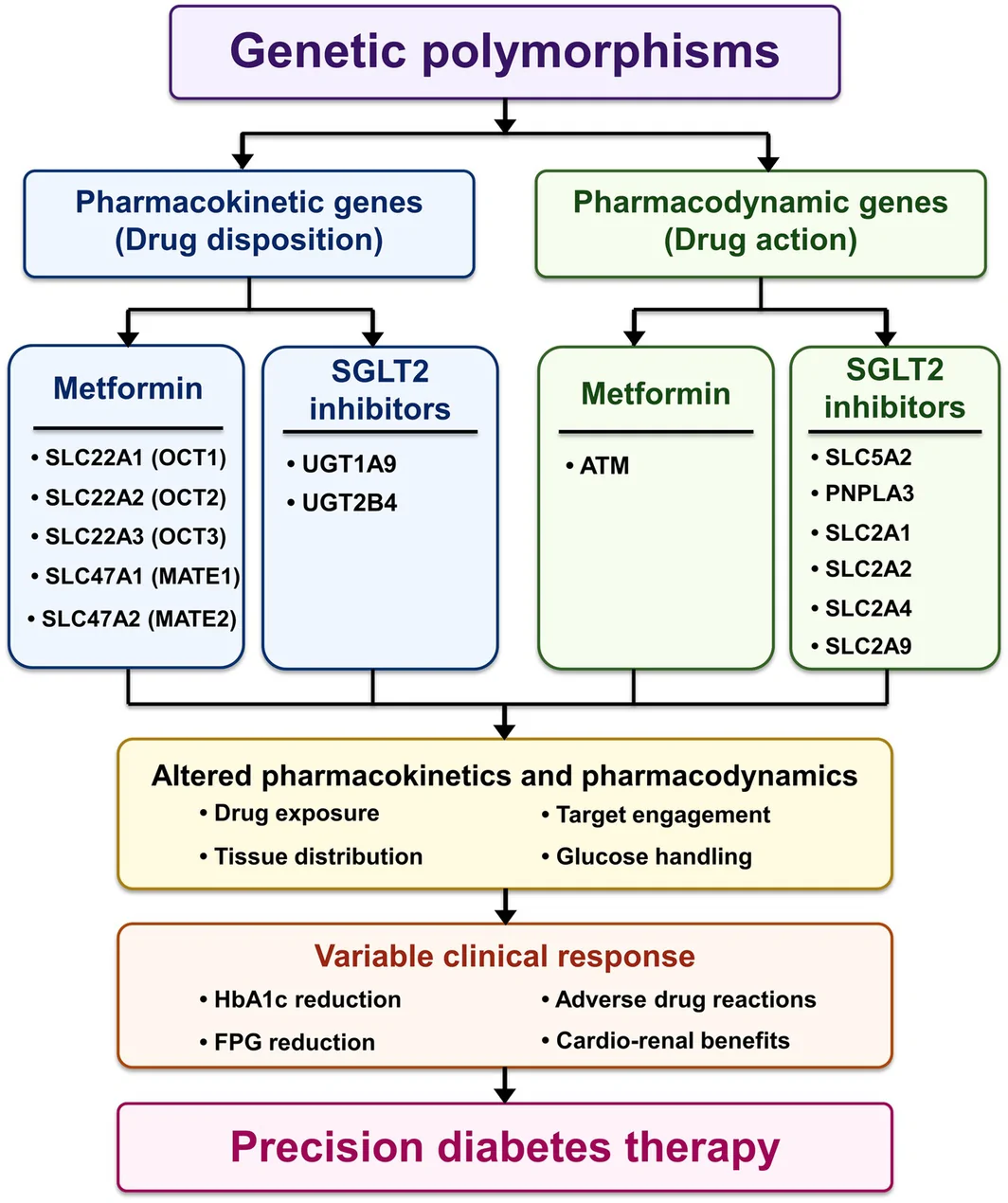
Integrated pharmacogenetic mechanisms underlying inter‐individual variability in metformin and SGLT2 inhibitor response. Genetic polymorphisms in pharmacokinetic (drug transport and metabolism) and pharmacodynamic (drug targets and signalling) genes alter drug exposure, tissue distribution, and target engagement, resulting in differences in glycemic efficacy, adverse drug reactions, and cardiorenal outcomes. These mechanisms provide the basis for precision diabetes therapy.

From a translational perspective, the available evidence supports the potential role of PGx in optimizing diabetes management while simultaneously highlighting the limitations that currently impede clinical implementation. Identification of patients carrying reduced‐function transporter variants or polymorphisms associated with diminished therapeutic response could facilitate earlier treatment optimization, improve glycemic control, reduce adverse drug reactions, and minimize prolonged exposure to ineffective therapies [46, 93]. However, despite substantial progress in identifying candidate pharmacogenetic markers, the existing evidence remains insufficient to support routine genotype‐guided prescribing. Most reported associations originate from relatively small studies, have not been consistently replicated across ethnically diverse populations, and frequently demonstrate modest effect sizes that limit their immediate clinical utility. Consequently, large prospective multicenter studies employing standardized phenotypic definitions, diverse populations, and robust validation strategies are essential before these genetic markers can be incorporated into evidence‐based clinical guidelines [6, 67].

Overall, this review demonstrates that pharmacogenetics has considerable potential to advance precision medicine in T2DM, although the current level of evidence varies substantially between drug classes. Metformin remains the most extensively investigated antidiabetic agent from a pharmacogenetic perspective, with consistent evidence supporting the contribution of transporter and signalling gene variants to therapeutic variability. In contrast, pharmacogenomic research involving SGLT2 inhibitors is still evolving, and currently available biomarkers require further validation before clinical application. Future studies should move beyond candidate‐gene approaches by integrating polygenic risk models with transcriptomic, metabolomic, proteomic and other multi‐omics data, together with advanced computational approaches, to improve prediction of therapeutic response. Such integrative strategies will be essential for translating pharmacogenomic discoveries into clinically actionable tools capable of supporting truly individualized diabetes management.

## 8 Future Directions

From the study of single‐gene PGx to the investigation of polygenic and multi‐omic approaches, the emerging domain of gene‐directed glycemic therapies will evolve in many key aspects that are likely to guide the development of future personalized therapy with metformin and SGLT2 inhibitors.

### 8.1 Polygenic Response Prediction

The focus of gene‐based PGx has so far been largely on identifying variants associated with drug response [102]. Future research must focus on the development of PRS relevant to metformin and SGLT2 inhibitors. The use of PRS in PGx research has already proven highly promising for enhancing drug response prediction; nevertheless, distinct challenges remain when developing PRS in the PGx‐GWAS setting, including the Eurocentricity of discovery cohorts, relatively small PGx sample sizes, and difficulty in differentiating between the effects of prognostics and predictors [103]. Particularly, multi‐trait PRS that account for genetic correlations among phenotypes [102].

### 8.2 Diversification of Study Populations

Eurocentric study populations used in existing pharmacogenomic studies severely limit the generalizability of current knowledge. Genetic susceptibility variants associated with T2DM and drug responses in South Asian, Latino and African individuals remain understudied compared to their counterparts in European populations [104]. Differences in metformin response exist between ethnic groups clinically and call for ethnicity‐based genetic architecture in order to enhance predictive modelling. In the absence of diverse ancestries in PRS discovery datasets, neither PRS model in the future will become relevant in clinical settings [105].

### 8.3 Integration of Multi‐Omics Data and Pharmaco‐Microbiomics

Genetic components are estimated to contribute to approximately 33% of individual variation in metformin response, with metformin metabolism and gut microbial diversity playing significant roles [106]. A pharmaco‐multi‐omics strategy that involves genomic medicine, proteomic medicine, metagenomics, metabolomics, transcriptomic medicine and epigenomics has been proposed recently [107]. The role of the gut microbiome in influencing metformin pharmacodynamics and glycaemic control as an active participant in pharmacodynamics deserves exploration when SGLT2 inhibitors are considered. This is due to the under‐studied area of how the microbiome interacts with SGLT2 inhibitors [108]. The use of AI technology in integrating data from multi‐omics platforms across multiple patient cohorts may be the most likely solution for achieving pharmacogenomic insights [109].

### 8.4 Implementation and Integration Into Electronic Medical Records (EMRs)

The practical challenges of integrating polygenic information into clinical practice are numerous as physicians have voiced their dissatisfaction with the commercial PGx panel test products that lack actionable information, are not integrated into the EMR, and do not include information about appropriate prescription. Efforts in the future should aim to develop efficient tools that allow for gene‐guided prescriptions of metformin and SGLT2 inhibitors using a decision support system embedded within the EMR [110].

### 8.5 Prospective Pharmacogenomics Studies

To fulfil the potential of precise glycemic management based on genomic data, prospective interventional pharmacogenomics studies, rather than retrospective observational studies, are necessary. Regarding metformin therapy, omics approaches that take advantage of lower cost sequencing and large biobanks may prove effective in prospectively identifying response and non‐response genotypes [50]. In the case of the SGLT2 inhibitors, pharmacogenomics studies incorporated into ongoing cardiovascular/renal trials appear to be the most economical approach to obtaining enough evidence to guide guidelines and practice [67].

## 9 Conclusion

Gene‐guided therapy represents a major step toward more precise and effective management of type 2 diabetes. Evidence from pharmacogenetic research suggests that inherited variation can influence individual responses to metformin and SGLT2 inhibitors, affecting both glycemic efficacy and treatment tolerability. The findings may contribute to more personalized approaches to therapy. Nevertheless, there is not enough evidence to apply the data in medical practice because the findings remain controversial and have to be validated on a large sample of subjects of different ethnicities. Future research is supposed to include genetic, clinical and environmental factors for better personalized medicine practices in patients suffering from type 2 diabetes.

## Author Contributions

**Imran Hossain:** conceptualization, methodology, data curation, validation, formal analysis, writing – original draft, supervision, investigation, writing – review and editing. **Mahnoor Haidar:** investigation, writing – review and editing, data curation, writing – original draft. **Mahfuj Ahmed Ratul:** investigation, data curation, writing – review and editing, writing – original draft. **Jannatul Ferdous Sumona:** investigation, data curation, writing – review and editing, writing – original draft. **Sraboni Shabnaz:** investigation, data curation, writing – review and editing, writing – original draft. **Abul Fojal Nokib:** investigation, data curation, writing – review and editing, writing – original draft, conceptualization, methodology, visualization, validation, project administration. **Fatema‐Tuz‐Zohora:** conceptualization, supervision, writing – review and editing.

## Funding

The authors have nothing to report.

## Conflicts of Interest

The authors declare no conflicts of interest.

## Data Availability

The data supporting the findings of this study can be obtained from the corresponding author upon reasonable request.
